# Associations of daily diet-related greenhouse gas emissions with the incidence and mortality of chronic diseases: a systematic review and meta-analysis of epidemiological studies

**DOI:** 10.4178/epih.e2023011

**Published:** 2022-12-30

**Authors:** Jee Yeon Hong, Young Jun Kim, Sanghyuk Bae, Mi Kyung Kim

**Affiliations:** 1 Department of Preventive Medicine, Hanyang University College of Medicine, Seoul, Korea; 2 Institute for Health and Society, Hanyang University, Seoul, Korea; 3 Department of Preventive Medicine, College of Medicine, The Catholic University of Korea, Seoul, Korea

**Keywords:** Systematic review, Meta-analysis, Greenhouse gases, Diet, Mortality

## Abstract

**OBJECTIVES:**

Although the entire process extending from food production to dietary consumption makes a large contribution to total greenhouse gas (GHG) emissions, little and inconsistent evidence exists on the epidemiological associations of daily diet-related GHG emissions with chronic disease risk or all-cause mortality. This systematic review and meta-analysis explored the observational epidemiological relationship between daily diet-related GHG emissions and health outcomes, including the risk of chronic diseases and all-cause mortality.

**METHODS:**

Original articles published in English until May 2022 were identified by searching PubMed, Ovid-Embase, Web of Science, CINAHL, and Google Scholar. The extracted data were pooled using both fixed-effects and random-effects meta-analyses and presented as hazard and risk ratios (RRs) with 95% confidence intervals (CIs).

**RESULTS:**

In total, 7 cohort studies (21 study arms) were included for qualitative synthesis and meta-analysis. The GHG emissions of dietary consumption showed a significant positive association with the risk of chronic disease incidence and mortality in both fixed-effects and random-effects models (fixed: RR, 1.04; 95% CI, 1.03 to 1.05; random: RR, 1.04; 95% CI, 1.02 to 1.06). This positive association was robust regardless of how daily diet-related GHG emissions were grouped. More strongly animal- based diets showed higher GHG emissions. However, there were only a few studies on specific chronic diseases, and the subgroup analysis showed insignificant results. There was no evidence of publication bias among the studies (Egger test: p=0.79).

**CONCLUSIONS:**

A higher GHG-emission diet was found to be associated with a greater risk of all-cause mortality.

## INTRODUCTION

Non-communicable diseases, including cardiovascular disease (CVD) and cancer, accounted for 73.4% of all deaths in 2017, representing 7.61 million additional deaths estimated in 2017 versus 2007 (a 22.7% increase) [1]. The link between the development of these chronic diseases and dietary risk factors is well known and has increasingly garnered attention. The Global Burden of Diseases study reported that in 2019, approximately 8.0 million deaths and 187.7 million disability-adjusted life-years were attributable to dietary risk factors [2], and a global transition to diets high in processed foods, refined foods such as sugars and fats, oils, and meats was identified as a major contributor [3-5].

Since 2019, climate change has caused forest fires, large-scale hurricanes, and tsunamis, resulting in massive casualties and the destruction of nature [6]. These abnormal weather conditions are mostly attributed to large-scale emissions of greenhouse gases (GHGs) [7]. These emissions not only increase the risk of climate disasters, such as abnormal heat waves and cold waves, but can also present new and diverse risks in various populations, such as those resulting from ecosystem destruction [7] and increased risks of chronic diseases [8,9].

Approximately one-quarter of climate change has been attributed to the process from food production to consumption, reaching 50% of total carbon emissions, especially when large amounts of carbon are emitted [10]. Furthermore, if unchecked, there will be an estimated 80% increase in global agricultural GHG emissions by 2050 [11]. Over the past decade, studies on health-related foods or dietary patterns that emit fewer GHGs have been actively conducted [12-17], with a particular focus on their associations with CVD [15-17]. In addition, several systematic reviews on diets with lower GHG emissions [18,19] or the relationships between diet-related GHG emissions and health outcomes, such as CVD [20], coronary heart disease [21,22], cancer [20-22], and death [20-24], have also been reported. However, those reviews were intended to theoretically model the reduction of diet-related GHG emissions or to present scenarios about the health consequences of consuming alternative foods with relatively low carbon emissions. To the best of our knowledge, no systematic review or meta-analysis has investigated the epidemiological associations of dietary patterns or food-related GHG emissions with the risk of chronic disease incidence, death from chronic diseases, and the risk of all-cause mortality.

We, therefore, aimed to conduct a systematic review and meta-analysis to evaluate whether an association exists between diet-related GHGs and the risks of all-cause mortality, chronic disease incidence, and mortality from chronic diseases.

## MATERIALS AND METHODS

### Data sources and searching strategy

This systematic review and meta-analysis followed the MetaAnalysis of Observational Studies in Epidemiology (MOOSE) guidelines [25]. We searched PubMed (https://www.ncbi.nlm.nih.gov/pubmed), Ovid-Embase (http://ovidsp.tx.ovid.com), Web of Science (https://www.webofscience.com), and CINAHL (https://www.ebsco.com) to identify published observational epidemiological studies through May 2022. Related keywords were set for PubMed, and other search engines were searched under the same conditions with slight modifications. We selected “food” and “diet,” which are basic terms from the highest level of the Medical Subject Headings (MeSH) tree (https://www.ncbi.nlm.nih.gov/mesh/), and “greenhouse gas” and “greenhouse effect,” which also are in the MeSH tree, to include all articles that estimated GHGs from dietary elements as the exposure keywords. For the outcome keywords, “chronic disease” and “mortality” were selected, which were the main outcomes of interest because they have been previously reported as major causes of death related to rises in temperature [8,9]. Our search strategy included those terms and the “all fields” filter with additional truncation options (Supplementary Material 1). The search filters for study design were provided by the University of Texas (https://libguides.sph.uth.tmc.edu/search_filters). In addition, a Google Scholar search was conducted to include papers that were not identified by searching the databases listed above or the latest papers.

### Inclusion/exclusion criteria and study selection

Studies were included if they (1) were original articles presenting observational epidemiological studies; (2) included adults aged 18 years or older without any sex restrictions; (3) estimated the amount of GHG emissions from daily dietary intake or food intake; (4) divided groups by the amount of GHG emissions or showed differences between groups in the amount of GHG emissions; (5) reported the incidence, prevalence, or mortality of chronic diseases such as CVD or cancer; and (6) were written in English. Using the Rayyan software (Qatar Computing Research Institute, Doha, Qatar) [26], duplicate articles were excluded, and then titles and abstracts were reviewed in the first exclusion process to remove articles not related to this study topic. We applied only 1 exclusion criterion (namely, a follow-up duration of less than 5 years in cohort studies), because the follow-up period required for a sufficient number of events has typically been at least 5 years in many prospective cohort studies [27]. In the second exclusion process, the full text was reviewed to select articles based on the inclusion and exclusion criteria. Two reviewers performed the process and were able to reach a consensus about the eligibility of all articles without requiring the involvement of a third investigator for resolution.

### Data extraction and study quality

The following relevant pieces of information were extracted: title; names of authors; country; year of publication; publication journal; objective; funding source and conflict of interest; study design (duration/follow-up period, population characteristics); basic information of the subjects (age range, sex distribution, sample size [the number of deaths or cases of total, comparison, and exposed participants], health status); exposure and comparison groups (amount of GHGs, method of calculation [diet assessment, unit reported, type of GHGs], and dietary pattern [when available]); type of outcomes; results from the adjusted model (risk ratio [RRs] or hazard ratios [HRs] and their 95% confidence intervals [CIs]); covariates; and conclusions.

When there were more than 2 groups, we used the lowest GHG emissions group as reference and the highest group as the comparison group [28]. In the opposite cases, we back-calculated the effect values by calculating the negative natural log and converting it using exponentiation [29].

The study quality was evaluated using the National Institutes of Health quality assessment tool for observational cohort studies (https://www.nhlbi.nih.gov/health-topics/study-quality-assessment-tools). A critical review was undertaken, and any disagreement was resolved by consensus.

### Statistical analysis

We extracted the RR or HR of the last adjusted model considering covariates [30]. Cases where the reference group was not shared by outcome were considered as a separate comparative analysis. For studies where the reference group was shared with more than 1 comparison or for studies testing GHGs from different dietary indices more than once in the same participants, we used the standard error, which was adjusted by the approximate adjustment method of multiplying the standard error by the square root of the ([number of study groups+1]/2) [31]. Heterogeneity was assessed with Cochran’s Q and the I² statistic [32] (p for difference < 0.1 [33]). However, to consider heterogeneous and common effects, both fixed-effect and random-effects models were used with the DerSimonian-Laird method for the pooled effect among studies [34]. A funnel plot was generated to assess the potential publication bias through a visual analysis, and the Egger regression asymmetry test was conducted at a significance level of 0.05.

Subgroup analyses were performed by (1) the type of outcome (all-cause mortality, CVD, cancer, and others) and (2) sex (all participants, males, and females). A sensitivity analysis was performed to evaluate the robustness of the meta-analytic results by removing 1 study arm at a time for all study arms (a method that is only applicable for meta-analyses with 10 or more study arms) and then recalculating the pooled effects. All analyses were conducted using R version 4.1.1 (R Foundation for Statistical Computing, Vienna, Austria), using the meta package.

### Ethics statement

As we used secondary data for systematic review and meta-analysis, therefore this article is eligible for institutional review board exemption (using data that already had approval).

## RESULTS

### Literature search results

**Figure 1.**
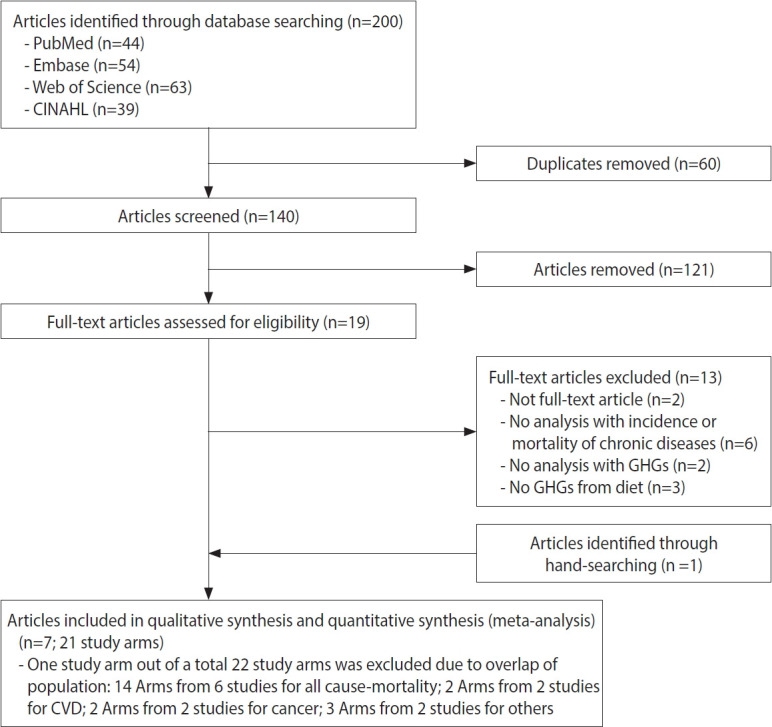
Flow chart of article selection for the association between GHG emissions from diet and disease mortality. GHG, greenhouse gas; CVD, cardiovascular disease.

In total, 140 articles (44 from PubMed, 54 from Embase, 63 from Web of Science, and 39 from CINAHL, with 60 duplicate records) that met the criteria were initially identified. After screening the titles and abstracts, 19 articles remained for full-text evaluation. One article was additionally identified through hand-searching in Google Scholar. Finally, 7 articles [12,35-40] were included in the qualitative systematic review and the meta-analysis. Among a total of 22 study arms, 1 study arm for all-cause mortality [12], which overlapped with another study arm conducted in the same population [38], was excluded. Finally, 21 study arms remained for the meta-analysis (Figure 1).

### Systematic review

#### Study design and participants

Study characteristics are presented in Table 1. Seven prospective cohort studies [12,35-40] were selected. Six studies were performed in Europe, and only 1 was conducted in the United States and Canadian populations [39]. Four European articles were from the European Prospective Investigation into Cancer and Nutrition (EPIC), with studies conducted in Spain [35] and the Netherlands (EPIC-NL) [12,38], or including the EPIC study (EPIC-Oxford in a multiple-cohort study [40]). In the multiple-cohort study using 3 cohorts [40], 2 cohorts with 5 follow-up years or longer were presented. The follow-up durations in the present study ranged from 5.8 years to 21.0 years and the age ranged from 20 years to 70 years. The ages of participants were 20 or higher in 3 studies [12,37,38], and 30 or higher in the rest of the studies, with a maximum of 70 years. With the exception of the Million Women Study (MWS), all cohorts included both males and females together, but findings in males and females were separately demonstrated in only 2 articles [36,38]. Those studies showed that males tended to have a higher amount of GHG emissions related to diet.

All 7 prospective cohort studies demonstrated an association between daily diet-related GHG emissions and all-cause mortality, but only 4 studies showed a positive association [35,38-40]. Two articles [12,35] evaluated the association of diet-related GHG emissions with the incidence and mortality risk of chronic diseases, such as CVD, cancer [12,35], type 2 diabetes [35], and respiratory disease [12]. However, only EPIC-Spain showed a significant positive association of GHG emissions with the incidence of coronary heart disease and type 2 diabetes [35].

#### GHG database and calculation of GHG emissions

Table 2 shows the GHG databases used to estimate the total amount of GHG emissions related to diet and how much food groups contributed to total GHG emissions. In all studies, total GHG emissions were estimated by weighing the amount of GHG per kilogram (CO_2_eq/day) while calculating the daily food consumption from food frequency questionnaires composed of 64 to 240 food items. GHG emissions in 4 studies [35-37,39] were adjusted for energy intake. The GHG emissions databases for foods were not identical: the GHG emissions database in the Swedish study [36] was provided by an independent, state-owned institute, the Research Institute of Sweden, and the 2 Dutch studies [12,38] used the database of an international research network, the Food Climate Research Network. Other studies used data from published articles [35,37,40]. Only 1 study mentioned that they imputed proxy values for 66 foods for which GHG values were not available from published articles [39]. However, all databases were based on a life cycle assessment (LCA), although 1 study did not include the packaging process [36]. Total GHG emissions in those databases were calculated using CO_2_, methane (CH4), and nitrous oxide (N2O). Generally, GHG emissions are calculated from production, and include processing and distribution, consumption, and waste.

The following formula is used:


∑s,iGHGi=Wi*emfi'

where denotes *GHG_i_* emissions from the total amount purchased of each food item, *W_i_* is the weight of each item (in kilograms) or volume in litres, and *emf_i_* is the emission factor associated with each item (emissions per kilogram or litre of item) [41]. The emission factors for each item were obtained from previous studies [42,43].

#### Contribution of food groups to total GHG emissions

Foods were classified into groups, ranging in number from 4 [40] to 19 [36], to calculate their proportional contribution to total GHG emissions (Table 2). The food groups that contributed the most to total GHG emissions differed depending on the method used to classify foods; for example, some studies [35,39,40] combined processed red meat and unprocessed red meat into one food group, but others [12,37] did not. Nevertheless, red meat seemed to be one of the food groups that contributed the largest proportion of total GHG emissions, while fruits and vegetables tended to have low contributions. Following red meat, milk and dairy products also made large contributions. The EPIC-Spain study [35] reported that 41.6% of GHG emissions were attributed to red meat. An EPIC-NL study [12] also showed that red meat had the highest contribution to total GHG emissions, accounting for almost 30% of total diet-derived GHG emissions. The other EPIC-NL study [38] considered 3 dietary indices (healthy diet indicator [HDI], Dietary Approaches to Stop Hypertension [DASH], and the Dutch healthy diet index 2015 [DHD15-index]). GHG emissions decreased significantly with increments in the scores of the HDI and DHD15-index, while there was no significant difference in GHG emissions between groups by DASH score. In the Adventist Health Study [39], the amount of GHG emissions from a vegetarian diet was lower than that of a non-vegetarian diet (2.16 vs. 3.05 kg CO_2_eq/day). The MWS [40] reported fixed GHG emissions values for the criteria in the Eatwell Guide recommendations, which show to what extent participants are achieving a healthy and balanced diet. The lowest-accordance group had the highest GHG emissions (5.4 kg CO_2_eq/day), and the highest-accordance group had the lowest GHG emissions (3.8 kg CO_2_eq/day).

### Meta-analysis

**Figure 2.**
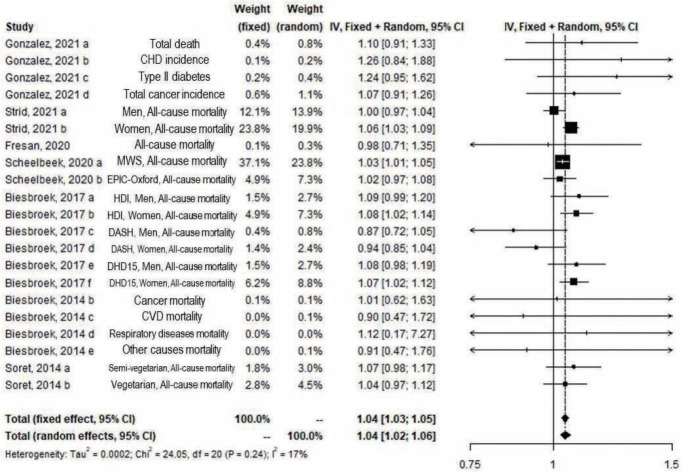
Forest plot of hazard ratios (risk ratio from Scheelbeek, 2020) and 95% confidence intervals (CIs) for greenhouse gas emissions from diet and disease incidence or mortality by outcome. The meta-analysis was undertaken using random-effects and fixed-effects models. CHD, coronary heart disease; MWS, Million Women Study; EPIC, European Prospective Investigation into Cancer and Nutrition; HDI, Healthy Diet Indicator; DASH, Dietary Approaches to Stop Hypertension; DHD15, Dutch Healthy Diet index 2015; CVD, cardiovascular disease; df, degree of freedom.

**Figure 3.**
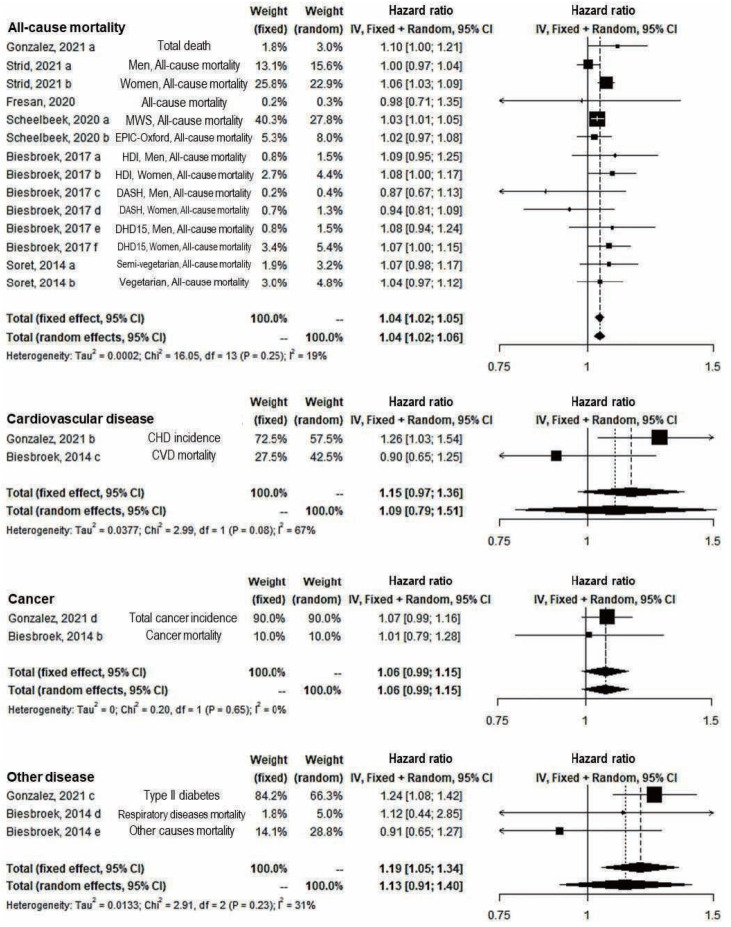
Subgroup analysis by the type of outcome of greenhouse gas emissions from diet and disease incidence or mortality (risk ratio from Scheelbeek, 2020). CI, confidence interval; MWS, Million Women Study; EPIC, European Prospective Investigation into Cancer and Nutrition; HDI, Healthy Diet Indicator; DASH, Dietary Approaches to Stop Hypertension; DHD15, Dutch Healthy Diet index 2015; CHD, coronary heart disease; CVD, cardiovascular disease; df, degree of freedom.

**Figure 4.**
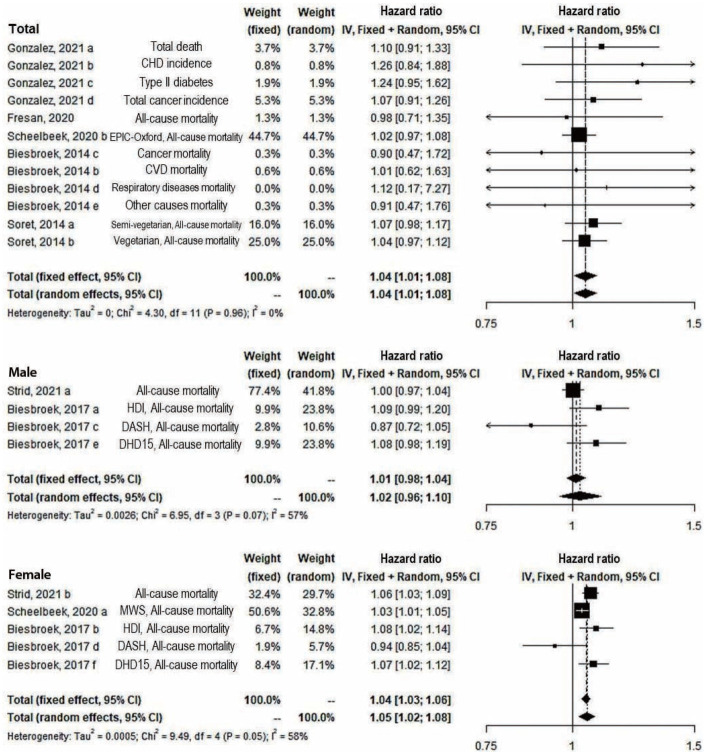
Subgroup analysis by sex of greenhouse gas emissions from diet and disease incidence or mortality (risk ratio from Scheelbeek, 2020). CI, confidence interval; CHD, coronary heart disease; EPIC, European Prospective Investigation into Cancer and Nutrition; CVD, cardiovascular disease; HDI, Healthy Diet Indicator; DASH, Dietary Approaches to Stop Hypertension; DHD15, Dutch Healthy Diet index 2015; MWS, Million Women Study; df, degree of freedom.

Figures 2-4 show the results of the overall meta-analysis and subgroup analyses by outcomes and sex. Significant results were shown in the overall meta-analysis, which included 7 studies with 756,966 participants and 21 study arms, in both the fixed-effect and random-effects models (fixed: RR, 1.04; 95% CI, 1.03 to 1.05; random: RR, 1.04; 95% CI, 1.02 to 1.06), with 14 arms from 6 studies for all cause-mortality, 2 arms from 2 studies for CVD incidence and mortality, 2 arms from 2 studies for cancer incidence and mortality, and 3 arms from 2 studies for the incidence and mortality of other conditions (Figure 2).

In the subgroup analysis of outcomes, the significant positive association remained only for all-cause mortality (14 study arms) (fixed: RR, 1.04; 95% CI, 1.02 to 1.05; random: RR, 1.04; 95% CI, 1.02 to 1.06) and the fixed effect model of other diseases’ incidence and mortality (3 study arms) (RR, 1.19; 95% CI, 1.05 to 1.34) (Figure 3). In both sexes (12 study arms) and only female (5 study arms), significant results were shown in both the fixed-effect and random-effects models (fixed and random: RR, 1.04; 95% CI, 1.01 to 1.08 in both sexes; fixed: RR, 1.04; 95% CI, 1.03 to 1.06; random: RR, 1.05; 95% CI, 1.02 to 1.08 in females). However, the results were not significant among males (Figure 4). In the sensitivity analysis for the overall effect using 21 study arms and for all-cause mortality regardless of diseases using 18 study arms, the overall effects appeared robust (data not shown).

### Publication bias and quality assessment

A funnel plot and the Egger test were used to identify potential publication bias (Supplementary Material 2). Even when the analysis was performed excluding 1 outlier [12] at the bottom of the funnel plot, the result of the meta-analysis was significant. There was no evidence of publication bias among the studies (Egger test: p= 0.79). For the quality assessment of the studies, we set an arbitrary standard and evaluated each study as “good” if there were at least 11 “yes” responses, “fair” if there were at least 7 and fewer than 11 “yes” responses, and “poor” if there were fewer than 7 “yes” responses. All studies were evaluated as “good” (Supplementary Material 3).

## DISCUSSION

The main findings in the present study were as follows: diets with higher GHG emissions were positively associated with the risk of chronic disease incidence and mortality, particularly in females, animal-based diets contributed most to diet-derived GHG emissions, and males tended to have diets with higher GHG emissions than females.

In the present study, the meta-analysis was conducted with a small number of articles and there were various countries, dietary surveys, and outcome variables. Therefore, a random-effects model analysis was also conducted to consider heterogeneity. However, we found about a 4% increase in both the fixed-effects and random-effects models for all-cause mortality in the highest group of diet-related GHG emissions, particularly in females. Based on the present study findings, the effect sizes for all-cause mortality did not seem to be heterogeneous. These findings were in accordance with the suggestions from modelling studies that a reduction of GHG emissions by dietary change could affect human health by about 1-16% [21,22], although there has been no review of climate impact on health in the real population.

There are 2 possible links between GHG emissions related to daily food consumption and health outcomes. The first is that foods that emit more GHGs were positively associated with chronic disease risk. The present study showed that red meat had the greatest influence on GHG emissions, followed by dairy products, seafood, and vegetables [12,35-40]. This finding was in accordance with a previous report in which red meat was demonstrated to emit about 150 times more GHGs than vegetable protein sources such as nuts and legumes, while dairy products emit 30 times to 40 times more than vegetable protein sources [11]. Because animal-based foods and their components increase the risk of many chronic diseases [44-49] and healthy dietary patterns such as the vegetarian and Mediterranean diets [11,50] mainly consist of plant-based foods, the positive association in the present study may also be associated with foods with high GHG emissions rather than the GHG itself. The second possible link is that increased dietary GHG emissions can affect everyone’s health through changing climate such as temperature, regardless of the amount of GHG emissions from the food sources consumed by a given individual. This possibility could not explain the present study’s finding of a positive association between high dietary GHG emissions and chronic disease outcomes, and there is no evidence whether climate change is more harmful to people with high consumption of animal-based foods [51]. Nevertheless, it may be important to consider previous evidence indicating that climate change, such as increased temperature, may result from massive GHG emissions and it may affect health conditions (dehydration, endothelial dysfunction) [44,45] and increase the risk of diseases (e.g., CVD, respiratory disease, cerebrovascular disease [46], and diabetes-related diseases) [47].

Diets with low GHG emissions are an important factor in establishing Sustainable Development Goals [11,52]. The findings related to diet-related GHG emissions should be interpreted carefully, because a plant-based diet is not always healthier and does not always emit lower GHGs (e.g., donuts) [53] and there are foods (e.g., coffee) that emit more GHGs than pork and chicken [54]. Moreover, seafood and vegetables also appear to emit high levels of GHGs in distribution and processing [55]. Additionally, high nutritional quality is not always associated with lower GHG emissions [20,21,24,56,57]. For example, sugar might have a lower environmental impact per calorie than other foods, and some fruits or vegetables such as lettuce, palm oil, and banana, have higher GHG emissions per calorie than dairy and non-ruminant meats [21,58,59].

Despite the finding that males usually consume a more animal-based diet, which emits more GHGs [60], we found no association between dietary GHG emissions and the risk of diseases in males, unlike in females. Unfortunately, there was no study with which to compare our findings in males. Furthermore, due to a lack of evidence in males and their very similar risk to that of females, we could not assert that a sex-based difference exists in the association of diet-related GHG emissions with all-cause mortality in the present study.

We explored not only the association between diet-derived GHGs and health, but also the methods to estimate GHG emissions in relation to diet. Only one study in Sweden used a stateowned database that did not include emissions from consumer transportation, storage, cooking, and waste management processes [36], while most other studies extracted data from previously published articles. As there is no standard GHG emissions database, the estimation of GHGs from food items remains unclear. Moreover, the LCA studies used in the articles reviewed in the present study reported GHG emissions based on their own selection of system boundaries and functional units [61,62], which might have substantially affected the estimated amount of GHG emissions for the same foods. Therefore, the study objectives should be considered when choosing the estimation method.

The critical limitation of this paper is the remarkably small number of papers estimating daily diet-related GHG emissions in the real population and their association with the mortality or incidence of chronic diseases, such as CVD and cancer. Due to a lack of articles, we could not draw firm conclusions regarding this association in the present study. The second limitation is that studies used various methods of GHG estimation, which may not be comparable, although significant results remained in a subgroup analysis according to the source of the GHG data (institution or literature review) (data not shown). A GHG database could contribute to the quality of estimated dietary GHG emissions, and it should be kept in mind that diet-related GHGs can be produced in various steps, such as food processing, transportation, manufacturing, consumption, and waste. However, although all studies except one [36] stated that they considered GHG emissions from production to consumption, none of the studies described the calculation method of each LCA step in detail and they did not mention the validity of their dietary assessment methods to estimate GHG emissions. Therefore, it may be important to evaluate and improve the GHG databases themselves and to validate dietary assessment methods in the future. Last, no study evaluated linear and non-linear dose-response relationships between GHG emissions and the risk of chronic diseases. Therefore, many issues remain to be studied.

Despite these limitations, it is worthwhile to note a positive association of diet-related GHGs with the risk of chronic disease incidence and mortality in the present study, because, to the best of our knowledge, the present study is the first systematic review and meta-analysis of the epidemiological association of daily dietderived GHG emissions with chronic disease incidence and mortality.

## CONCLUSION

The present study finding suggested that the relative emissions of diet-related GHGs were positively associated with the risk of chronic disease incidence and mortality, and there is a possibility that healthy food choices contribute to reducing diet-derived GHG emissions.

## DATA AVAILABILITY

The data that support the findings of this study are available from the corresponding author, upon reasonable request.

## Figures and Tables

**Table 1.** Characteristics of observational studies included in the systematic review and meta-analysis of GHG emissions from diet and the risk of chronic disease incidence and mortality

Author, year, country	Studydesign	Cohort, population (n)	Dietary intake assessment	Grouping of GHG emissions; reference group for comparison	GHG amount (kg CO_2_eq/day) in lowest vs. highest group	Outcome	Follow-up (yr)	No. of deaths/cases in the total study population and the lowest and highest groups	Lowest vs. highest reported outcome, HR (95% CI)	Study arms	Adjustment variables
González, 2021, Spain [35]	Prospective cohort study	EPIC-Spain	Dietary history questionnaire (240 Spanish food list)	Tertiles of calculated GHG emissions of diet; T1	Tertile cut-off <2.53 vs. >3.25	Total death	Total deaths 18.00; CHD 10.40; T2D 12.10, total cancer 12.10	3,561/1,173/1,285	1.10 (1.01, 1.20)	a	Age, sex, centre
		n=40,621 (M: 15,323, F: 25,298)									
												Age 30-70 yr	CHD incidence		1,005/300/378	1.26 (1.08, 1.48)	b
		T2D incidence					2,025/625/775	1.24 (1.11, 1.38)	c			
		Total cancer incidence					4,457/1,486/1,590	1.07 (0.99, 1.15)	d			
		Strid, 2021, Sweden [36]				Prospective cohort study	VIP	64-FFQ	High nutrient density group	Group median		All-cause mortality	M: 16.00	M, 2,179/874/1,305	M: 1.00 (0.96, 1.03)	a	Age, age squared, BMI, physical activity, educational level, smoking status, and year of participation
n=51,432 (M: 25,438, F: 25,994)	M: 3.1 vs. 4.4		F: 14.70	W, 1,609/744/865	F: 1.06 (1.02, 1.11)		b										
Age 35-65 yr	F: 2.6 vs. 3.5																
Fresán, 2020, Spain [37]	Prospective cohort study	SUN Cohort	136-item FFQ	Quartiles of calculated GHG emissions of diet; Q1	Quartile mean 2.25 vs. 4.97	All-cause mortality	12.25	305/87/75	0.98 (0.71, 1.35)		Age, sex, BMI, adding a quadratic term, smoking, physical activity, time watching television, marital status, hypercholesterolemia, and hypertension
		n=17,387 (M: 6,781, F: 10,606)									
		Age 37±12 yr									
Scheelbeek, 2020, Europe [40]	Multiple cohort study	Cohort 1: MWS	130-item semi-quantitative questions	Tertiles of EWG accordance; T1 (the high EWG accordance group=the lowest GHG emissions group)	Tertile mean 3.8 vs. 5.4	All-cause mortality	10.50	33,531/7,864/9,925	RR: 1.03 (CI 1.01, 1.05)	a	Sex, region, method of recruitment, smoking, deprivation, alcohol consumption, height, BMI, exercise levels, hormone replacement therapy use, education, high blood pressure or hypertension, and energy intake
		n=464,078 (M: 0, F: 464,078)									
		Age average 56 yr									
		Cohort 2: EPIC-Oxford	130-item semi-quantitative FFQ				21.00	3,230/1,037/867	RR: 1.02 (0.97, 1.08)	b	
		n=40,030 (M: 9,607, F: 30,423)									
		Age average 56 yr									
		Follow-up									
Biesbroek, 2017, Netherlands [38]	Prospective sub-cohort study	EPIC-NL	178-item FFQ	Tertiles of HDI, DASH, DHD15-index; T3 (the lowest GHG emissions group)	Tertile mean (HDI)	All-cause mortality	19.20	M: 892/292/292	M: 1.09 (1.01, 1.17)	a	Age, BMI, educational level, smoking status, total daily energy intake, physical activity level, alcohol intake
		n=35,031 (M: 9,213, F: 25,818)			M: 4.42 vs. 4.87			F: 2,954/875/1,022	F: 1.08 (1.04, 1.13)	b	
		Age 20-70 yr			F: 3.66 vs. 3.83						
					Tertile mean (DASH)			M: 891/338/259	M: 0.87 (0.74, 1.04)	c	
					M: 4.59 vs. 4.62			F: 2,954/980/1,074	F: 0.94 (0.86, 1.03)	d	
					F: 3.68 vs. 3.76						
					Tertile mean (DHD15)			M: 891/293/269	M: 1.08 (1.01, 1.17)	e	Age, BMI, educational level, smoking status, total daily energy intake, physical activity level
					M: 4.48 vs. 4.74			F: 2,954/1,000/990	F: 1.07 (1.03, 1.11)	f	
					F: 3.63 vs. 3.82						
Biesbroek, 2014, Netherlands [12]	Prospective cohort study	EPIC-NL	178-item FFQ	Quartiles of GHG; lowest GHG quartile	Quartile median 2.86 vs. 5.12	All-cause mortality	15.90	2,563/736/570	0.95 (0.77, 1.15)	a	Age, sex, energy intake
		n=35,079 (M: 9,401, F: 25,678)				Cancer mortality		1,193/324/268	1.01 (0.86, 1.34)	b	
		Age 20-70 yr				CVD mortality		545/164/120	0.90 (0.63, 1.28)	c	
						Respiratory diseases mortality		137/41/27	1.12 (0.52, 2.39)	d	
						Other-cause mortality		529/157/120	0.91 (0.64, 1.30)	e	
Soret, 2014, USA and Canada [39]	Prospective cohort study	AHS2	Self-administered 210-item FFQ	Semi-vegetarian vs. Non-vegetarian	Group mean (n=28,888)	All-cause mortality	5.79	987/410/577	1.07 (1.02, 1.12)	a	Age, sex, race, smoking, exercise, personal income, educational level, marital status, alcohol, region, and sleep, menopause, and hormone therapy
		n=73,308 (M: 25,105, F: 48,203)			2.39 vs. 3.05						
				Vegetarian vs. Non-vegetarian	Group mean (n=44,420)			1,583/1,006/577	1.04 (1.00, 1.08)	b	
		Age average 56.8 yr			2.16 vs. 3.05						

GHG, greenhouse gas; HR, hazard ratio; CI, confidence interval; M, male; F, female; CHD, coronary heart disease; T2D, type 2 diabetes; CO_2_, carbon dioxide; CH4, methane; N2O, nitrous oxide; kg CO_2_eq/day, CO_2_ equivalent kilograms per day; LCA, life cycle assessment; RR, risk ratio; FFQ, food frequency questionnaire; RISE, Research Institutes of Sweden; BMI, body mass index; HDI, Healthy Diet Indicator; DASH, Dietary Approaches to Stop Hypertension; DHD15, Dutch Healthy Diet index 2015; CVD, cardiovascular disease; NDNS, National Diet and Nutrition Survey; EWG, Eatwell Guide; RR, risk ratio; NA, not available; AHS 2, Adventist Health Study 2; EPIC, European Prospective Investigation into Cancer and Nutrition; NL, Nether lands; MWS, Million Women Study; SUN, Seguimiento Universidad de Navarra; VIP, Västerbotten Intervention Programme.

**Table 2.** GHG databases, GHG estimation method, and food items used to calculate GHG emissions of daily food consumption

Author, year, country	Diet data	GHG database of foods used to estimate total GHG emission					The no. of foods finally used to estimate total GHG	Total energy adjusted GHGs from food intake (yes/no/NA)	The no. of the food groups/their contributions (%) to total GHG emissions
		Provided database	Data from the literature	Imputation	Type of GHGs	Unit			
González, 2021, Spain [35]^1^	240-food list		Yes		CO_2_, CH_4_, N_2_O	kg CO_2_eq/day	57 Food items	Yes	9 Food groups/red and processed meat: 41.59%; dairy products: 19.02%; other: 12.74%; fish and mollusks: 9.15%; fruit: 4.20%; poultry: 3.73%; vegetables: 3.40%; cereals: 2.69%; eggs: 2.48%; legumes: 0.99%
Strid, 2021, Sweden [36]^2^	64-item FFQ	RISS: an independent, state-owned institute			NA	kg CO_2_eq/day	57 Food items	Yes	19 Food groups/ NA
Fresán, 2020, Spain [37]^3^	136-item FFQ		Yes		CO_2_, CH_4_, N_2_O	kg CO_2_eq/day	94 Food items	Yes	14 Food groups (dairy products: 2.99 kg CO_2_eq/day; eggs: 2.74; vegetables: 2.67; fresh fruit: 2.37; processed meat: 2.08; oils and fats: 1.90; cereals: 1.89; white meat: 1.02; pastry products: 1.01; fish and seafood: 0.72; legumes: 0.38; red meat: 0.31; nuts: 0.15; processed fruit: 0.11)/NA
Scheelbeek, 2020, Europe [40]^4^	158 Distinct food groups from the National Diet and Nutrition Survey		Yes		CO_2_, CH_4_, N_2_O	kg CO_2_eq/day	NA	No	4 EWG recommendation groups (red and processed meat: -1.48 kg CO_2_eq/day; oily fish: 0.18 kg CO_2_eq/day; non-oily fish: 0.34 kg CO_2_eq/day; fruit and vegetables: 0.34 kg CO_2_eq/day)/NA
Biesbroek, 2017, Netherlands [38]^1^	178-item FFQ	FCRN based at the University of Oxford			CO_2_, CH_4_, N_2_O	kg CO_2_eq/day	NA	NA	NA
Biesbroek, 2014, Netherlands [12]^1^	178-item FFQ	FCRN based at the University of Oxford			CO_2_, CH_4_, N_2_O	kg CO_2_eq/day	NA	NA	21 Food groups/non-processed meat: 25.7%; cheese: 11.6%; milk: 9.5%; non-alcoholic: 9.4%; fruit, nuts and seeds: 5.6%; processed meat: 5.6%; vegetables: 5.5%; milk-based desserts: 4.1%; bread products: 3.4%; alcohol: 3.4%; sugar and confectionary: 2.5%; fat: 2.3%; fish: 2.1%; cake and biscuits: 2.1%; miscellaneous: 2.1%; potatoes: 1.9%; pasta, rice and couscous: 1.5%; egg: 1.2%; condiments and sauces: 0.8%; soups: 0.6%; legumes: 0.3%
Soret, 2014, USA and Canada [39]^5^	Self-administered 210-item FFQ		Yes	Using proxy values for 66 foods	CO_2_, CH_4_, N_2_O	kg CO_2_eq/day	210 Food items	Yes	5 Food groups:
									- Non-vegetarian total 3.05 kg CO_2_eq/day (plant foods: 40%; meat: 20%; dairy and eggs: 19%; beverages: 17%; other foods: 4%)
									- Semi-vegetarian total 2.39 kg CO_2_eq/day (plant foods: 59%; dairy and eggs: 17%; beverages: 14%; meat: 6%; other foods: 4%)
									- Vegetarian total 2.16 kg CO_2_eq/day (plant foods: 68%; dairy and eggs: 15%; beverages: 13%; other foods: <4%; meat: 0%)

All databases were estimated based on a LCA. GHG, greenhouse gas; FFQ, food frequency questionnaire; CO_2_, carbon dioxide; CH4, methane; N2O, nitrous oxide; kg CO_2_eq/day, CO_2_ equivalent kilograms per day; RISS, Research Institute of Sweden; FCRN, Food Climate Research Network database; LCA, life cycle assessment; EWG, Eatwell Guide; NA, not available; NDNS, National Diet and Nutrition Survey; DASH, Dietary Approaches to Stop Hypertension.

1 Contribution of different food groups to daily intake and GHG emissions (CO_2_eq (%))

2 Accurate information of contribution of food/food group was not available due to legal restrictions.

3 Mean value of energy-adjusted GHG emissions (kg CO_2_eq/day)

4 Used dietary data of NDNS and gave the fixed GHG emission values for the EWG criteria, compared groups by subtracting meeting EWG recommendations from not meeting EWG recommendations.

5 Comparison of GHG emissions (kg CO_2_eq/day) by major food groups and dietary pattern, adjusted to 2,000 kcal.

## SUPPLEMENTARY MATERIALS

Supplementary Material 1.

Supplementary Material 2. Funnel plot for the GHG emission from diet and chronic disease incidence and mortality.

Supplementary Material 3. The National Institutes of Health (NIH) quality assessment tool for observational cohort and cross-sectional studies.
