# Supplementary material for: Associations of daily diet-related greenhouse gas emissions with the incidence and mortality of chronic diseases: a systematic review and meta-analysis of epidemiological studies
Source: Epidemiol Health. 2022 Dec 30;45:e2023011. doi: 10.4178/epih.e2023011 (PMC10581893; doi:10.4178/epih.e2023011)
Supplement: Supplementary Material 1. [file epih-45-e2023011-Supplementary-1.docx]

Supplementary Materials: Associations of daily diet-related greenhouse gas emission with chronic diseases incidence and mortality: a systematic review and meta-analysis of epidemiological studies

Supplementary Material 1

(a) Search Terms used for systemic review and meta-analysis (PubMed).

| TERM | |
| --- | --- |
| Exposure | (Diet*) OR (Diet) OR (Food*) OR (Food) |
|  | (Greenhouse Gas) OR (Greenhouse Gases) OR (greenhouse effect) OR (GHG) OR (GHGs) OR (greenhouse gas emission) |
| Outcome | (Chronic Disease) OR (Chronic Disease*) OR (Mortality) OR (Mortalit*) OR (Cardiovascular Diseases) OR (Cardiovascular Disease*) OR (CVD) OR (Cancer) |
| Study Design | cohort studies[mesh:noexp] OR longitudinal studies[mesh:noexp] OR follow-up studies[mesh:noexp] OR prospective studies[mesh:noexp] OR retrospective studies[mesh:noexp] OR cohort[TIAB] OR longitudinal[TIAB] OR prospective[TIAB] OR retrospective[TIAB] |
|  | “Case-Control Studies”[Mesh:noexp] OR "retrospective studies"[mesh:noexp] OR “Control Groups”[Mesh:noexp] OR (case[TIAB] AND control[TIAB]) OR (cases[TIAB] AND controls[TIAB]) OR (cases[TIAB] AND controlled[TIAB]) OR (case[TIAB] AND comparison*[TIAB]) OR (cases[TIAB] AND comparison*[TIAB]) OR “control group”[TIAB] OR “control groups”[TIAB] |
|  | Cross-Sectional Studies[Mesh:noexp] OR cross-sectional[TIAB] OR Prevalence[mesh:noexp] OR prevalence[tiab] OR transversal study[tiab] |
|  | Incidence[mesh:noexp] OR incidence[tiab] |
|  | “Epidemiologic Studies”[Mesh:noexp] |

(b) Search Terms used for systemic review and meta-analysis (EMBASE).

| TERM | |
| --- | --- |
| Exposure | Diet*.mp. OR Food*.mp. |
|  | Greenhouse Gas*.mp. OR greenhouse effect.mp. OR GHG*.mp. OR greenhouse gas emission.mp. |
| Outcome | Chronic Disease*.mp. OR Mortalit*.mp. OR Cardiovascular Disease*.mp. OR CVD.mp. OR Cancer*.mp. |
| Study Design | cohort studies/ or longitudinal studies/ or follow-up studies/ or prospective studies/ or retrospective studies/ or cohort.ti,ab. or longitudinal.ti,ab. or prospective.ti,ab. or retrospective.ti,ab. |
|  | Case-Control Studies/ or Control Groups/ or Matched-Pair Analysis/ or ((case* adj5 control*) or (case adj3 comparison*) or control group*).ti,ab. |
|  | Cross-Sectional Studies/ or Prevalence/ or (cross-sectional or prevalence or transversal).ti,ab,kw. |
|  | Incidence/ or incidence.ti,ab,kw. |
|  | Epidemiologic Studies/ |

(c) Search Terms used for systemic review and meta-analysis (Web of Science).

| TERM | |
| --- | --- |
| Exposure | ALL=(Diet* OR Food*) |
|  | ALL=(Greenhouse Gas* OR greenhouse effect OR GHG* OR greenhouse gas emission) |
| Outcome | ALL=(Chronic Disease* OR Mortalit* OR Cardiovascular Disease* OR CVD OR Cancer*) |
| Study Design | ALL=(cohort studies OR longitudinal studies OR follow-up studies OR prospective studies OR retrospective studies) OR TI=(cohort OR longitudinal OR prospective OR retrospective) OR AB=(cohort OR longitudinal OR prospective OR retrospective) |
|  | ALL=(Case-Control Studies OR Control Groups OR retrospective studies) OR TI=((case AND control) OR (cases AND controls) OR (cases AND controlled) OR (case AND comparison*) OR (cases AND comparison*) OR control group OR control groups) OR AB=((case AND control) OR (cases AND controls) OR (cases AND controlled) OR (case AND comparison*) OR (cases AND comparison*) OR control group OR control groups) |
|  | ALL=(Cross-Sectional Studies OR Prevalence) OR TI=(cross-sectional OR prevalence OR transversal study) OR AB=(cross-sectional OR prevalence OR transversal study) |
|  | ALL=(Incidence) OR TI=(Incidence) OR AB=(Incidence) |
|  | ALL=(Epidemiologic Studies) OR TI=(Epidemiologic Studies) OR AB=(Epidemiologic Studies) |

(d) Search Terms used for systemic review and meta-analysis (CINAHL).

| TERM | |
| --- | --- |
| Exposure | (TX (Diet* OR Food*) |
|  | (TX (Greenhouse Gas* OR greenhouse effect OR GHG* OR greenhouse gas emission)) |
| Outcome | (TX (Chronic Disease* OR Mortalit* Cardiovascular Disease* OR CVD OR Cancer*)) |
| Study Design | (MH (cohort studies)) OR (MH (longitudinal studies)) OR (MH (follow-up studies)) OR (MH (prospective studies)) OR (MH (retrospective studies)) OR (TI (cohort)) OR (TI (longitudinal)) OR (TI (prospective)) OR (TI (retrospective)) OR (AB (cohort)) OR (AB (longitudinal)) OR (AB (prospective)) OR (AB (retrospective)) |
|  | (MH (“Case-Control Studies”)) OR (MH ("retrospective studies")) OR (MH (“Control Groups”)) OR (TI (case AND control) OR (TI (cases AND controls) OR (TI (cases AND controlled) OR (TI (case AND comparison*)) OR (TI (cases AND comparison*)) OR (TI (“control group”)) OR (TI (control groups)) OR (AB (case AND control)) OR (AB (cases AND controls)) OR (AB (cases AND controlled)) OR (AB (case AND comparison*)) OR (AB (cases AND comparison*)) OR (AB (“control group”)) OR (AB (control groups)) |
|  | (MH (Cross-Sectional Studies)) OR (MH (Prevalence)) OR (TI (cross-sectional)) OR (TI (prevalence)) OR (TI (transversal study)) OR (AB (cross-sectional)) OR (AB (prevalence)) OR (AB (transversal study)) |
|  | (MH (Incidence)) OR (TI (incidence)) OR (AB (incidence)) |
|  | (MH (“Epidemiologic Studies”)) |
