# Supplementary material for: Associations of daily diet-related greenhouse gas emissions with the incidence and mortality of chronic diseases: a systematic review and meta-analysis of epidemiological studies
Source: Epidemiol Health. 2022 Dec 30;45:e2023011. doi: 10.4178/epih.e2023011 (PMC10581893; doi:10.4178/epih.e2023011)
Supplement: Supplementary Material 2. — Funnel plot for the GHG emission from diet and chronic disease incidence and mortality. [file epih-45-e2023011-Supplementary-2.docx]

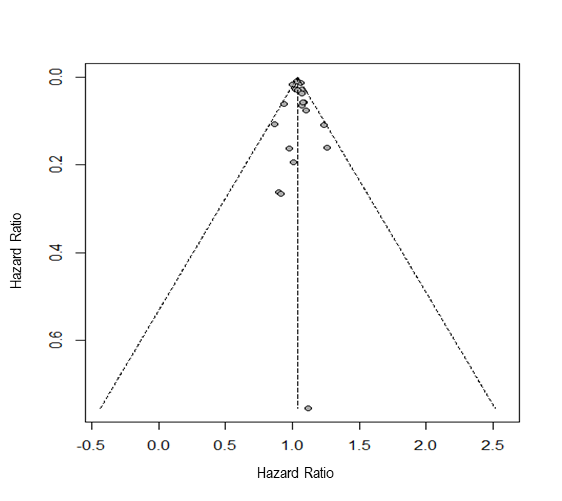


Supplementary Material 2. Funnel plot for the GHG emission from diet and chronic disease incidence and mortality.
